# Case Report: Multidrug Resistant *Raoultella ornithinolytica* in a Septicemic Calf

**DOI:** 10.3389/fvets.2021.631716

**Published:** 2021-03-26

**Authors:** Mathilde L. Pas, Kevin Vanneste, Jade Bokma, Laura Van Driessche, Sigrid C. J. De Keersmaecker, Nancy H. Roosens, Freddy Haesebrouck, Filip Boyen, Bart Pardon

**Affiliations:** ^1^Department of Large Animal Internal Medicine, Faculty of Veterinary Medicine, Ghent University, Merelbeke, Belgium; ^2^Sciensano, Transversal Activities in Applied Genomics, Brussels, Belgium; ^3^Department of Pathology, Bacteriology and Avian Diseases, Faculty of Veterinary Medicine, Ghent University, Merelbeke, Belgium

**Keywords:** antimicrobial resistance, *Enterobacteriaceae*, case report, cattle, sepsis, whole genome sequencing

## Abstract

Sepsis is a frequent life-threatening condition in young calves, requiring rapid broad spectrum and bactericidal therapy to maximize survival chances. Few studies have identified and characterized bacteria involved in sepsis in calves. This report demonstrates the involvement of a multidrug resistant *Raoultella ornithinolytica*, an emerging pathogen in human medicine, in a calf with suspected sepsis. *R. ornithinolytica* was identified by MALDI-TOF MS from blood cultures of a critically ill calf. Susceptibility testing showed phenotypic resistance against ampicillin, gentamicin, potentiated sulphonamides, streptomycin, tetracyclines and intermediate susceptibility for enrofloxacin. Whole genome sequencing confirmed identification as *R. ornithinolytica* and the multidrug resistant character of the isolate. Antimicrobial resistance genes acting against aminoglycosides, beta-lactam antibiotics, fosfomycin, quinolones, sulphonamides, trimethoprim and tetracyclines were found. The calf recovered after empirical parenteral therapy with enrofloxacin and sodium penicillin for seven days. Ancillary therapy consisted of fluid therapy, ketoprofen and doxapram hydrochloride. To the authors' knowledge, this is the first report characterizing a multidrug resistant *R. ornithinolytica* isolate from blood culture in cattle. It is currently unknown whether animals and farms may act as reservoirs for multidrug resistant *R. ornithinolytica* strains.

## Introduction

Sepsis is a high mortality risk disease for which rapid appropriate antibiotic treatment is critical to increase survival rates, both in cattle and in humans (1, 2). A multitude of bacteria have been described as causative agents, but in cattle mainly *Enterobacteriaceae*, more specifically *Escherichia coli*, are typically isolated from ill calves (3). Here, a multidrug resistant *Raoultella ornithinolytica*, another member of the *Enterobacteriaceae* family, isolated from a calf with neonatal septicemia, is described.

Multidrug resistant *R. ornithinolytica* strains have been reported in septicemia cases in humans, but not in cattle (4). *Raoultella ornithinolytica* is an encapsulated Gram-negative bacterium, until 2001 classified within the *Klebsiella* genus, reflecting that identification of this species using only conventional biochemical methods can be challenging (5). The genus *Raoultella* contains four species, of which *R. ornithinolytica* is described as the most virulent species in humans (6). Aside from its ubiquitous presence in aquatic environments (4, 6, 7), *R. ornithinolytica* is an opportunistic pathogen mainly involved in hospital-acquired infections, often after invasive procedures and in immunocompromised patients (4, 5, 7). This species has been linked with bacteremia, biliary tract and urinary tract infections, as well as pancreatitis and wound infections in humans (4, 6–8). To the best of our knowledge, septicemia caused by *R. ornithinolytica*, has not been described in other mammals. Though the veterinary importance of this bacterium seemed limited in the past, it has been isolated in feces of fish, ticks, termites, birds, pigs and turtles, as well as a case of pneumonia from the latter species (9–13). In cattle, *Raoultella* species have been isolated from rumen and manure samples (14). *Raoultella ornithinolytica* was also associated with mastitis and isolated from the viscera of a neonatal calf on autopsy with signs suggestive for septicemia (15, 16). In this study, *R. ornithinolytica* was isolated from a blood culture of a calf with suspected sepsis. Additionally, the genome sequence of this bacterium and antimicrobial resistance were determined.

## Case Description

A 2-day-old male Belgian blue calf (45 kg) was presented in December 2018 in a critically ill state. The calf had experienced breathing difficulties since the first day after birth. The calf had been treated by the local practitioner with furosemide, amoxicillin, dexamethasone, alpha tocopherol acetate and anhydrous sodium selenite, presumably all intramuscularly as indicated by the manufacturer, except for the furosemide, which was likely given intravenously. At presentation, the calf was in lateral decubitus and depressed. Fever (40.8°C: ref. 38.5–39.5) was present, with an increased respiratory rate (72/min: ref. 20–50), but heart rate (108/min: ref. 90–110) was normal (17). Ultrasonography was performed with a linear 7.5 MHz probe (Easote MyLab™30 Gold unit, the Netherlands) and revealed diffuse comet tail artifacts (B-lines) on the pleura and multiple small consolidations (max. 1 cm depth) on both sides of the thorax. No abnormalities were found on ultrasonographic examination of the abdomen. Blood samples were collected from the jugular vein upon arrival and placed in heparin-coated tubes prior to blood-gas analysis with RAPIDPoint® 405 (Siemens Healthcare, Beersel, Belgium). Venous blood gas analysis showed a mild metabolic acidosis (pH 7.23). The morning after presentation, an arterial sample out of the external carotid artery displayed hypoxemia [arterial pO_2_ = 32.6 mmHg (ref. 80–100)] and hypercapnia [arterial pCO_2_ = 74.7 mmHg (ref. 40–50)]. Failure of passive transfer of immunoglobulins was excluded with a glutaraldehyde test according to Turgut et al. (18).

## Diagnostic Assessment

### Bacteriology

Blood samples were aseptically sampled in one single volume collected from the jugular vein upon arrival, whereby 3 and 10 ml were aseptically inoculated into BD BACTEC™ Peds Plus™ and BD BACTEC™ Plus Aerobic medium, respectively (BD, Erembodegem, Belgium), which were subsequently incubated at 35°C in an automated system for the detection of microbial growth (BACTEC™ FX). For identification, a loopful of medium was first plated on a Columbia agar supplemented with 5% sheep blood (blood agar; Oxoïd, Hampshire, UK) and incubated for 24 h at 37°C in a 5% CO_2_ enriched atmosphere, after which a single colony was transferred on a polished steel plate, air dried and covered with 1 μL alpha-cyano-4-hydroxycinnamic acid matrix (Bruker Daltonics, Bremen, Germany). The sample was identified with an Autoflex III smartbeam MALDI-TOF MS, using FlexControl and MBT Compass software (Bruker Daltonics, Bremen, Germany). Log score values higher than 2.00, between 1.70 and 1.99, and between 0 and 1.69, indicate high-confidence, low-confidence, and no-confidence identification, respectively. From both BD BACTEC™ media (Becton Dickinson—Benelux, Erembodegem, Belgium), a pure culture was obtained. The isolate was identified with MALDI-TOF MS, and the best hit was obtained for *R. ornithinolytica* MB_18887 CHB with a (log)score value of 2.32. The seven best hits matched with *R. ornithinolytica*, with (log)score values ≥2.16.

Antimicrobial susceptibility testing (AST) for different antibiotics (Table 1) was executed with the disk diffusion test using a Mueller Hinton agar and aerobic incubation at 35°C for 20 h after which the inhibition zones were interpreted. The diameter of the inhibition zone was measured and interpreted according to the breakpoints prescribed by the Clinical and Laboratory Standards Institute (CLSI) (19). CLSI breakpoints specifically for *R. ornithinolytica* were not available, so CLSI values were used for Enterobacterales, since *R. ornithinolytica* belongs to the *Enterobacteriaceae* family. For ceftiofur, cattle-specific disk diffusion CLSI breakpoints were available. For the other antibiotics, CLSI breakpoints extrapolated from human breakpoints were used, rather than extrapolation from other organisms. In the absence of CLSI values for Enterobacterales for certain antibiotics (or certain potencies), breakpoints provided by the manufacturer were used (20) (Table A1 in Appendix). *Escherichia coli* strains ATCC® 25922™ and ATCC® 35218™ were used as quality control strains. Multidrug resistance was defined as resistance to agents from at least three different antimicrobial classes (21). The isolate showed multidrug resistance with the disk diffusion test, with resistance against ampicillin, tetracycline and doxycycline, gentamicin, potentiated sulfonamides and streptomycin and intermediate susceptibility for enrofloxacin (Table 1 and Table A1 in Appendix).

**Table 1:** Antibiotic susceptibility and antimicrobial resistance genes of *Raoultella ornithinolytica* obtained from a calf with sepsis.

**Antimicrobial**	**Antimicrobial susceptibility (disk diffusion)**	**Antimicrobial resistance genes**				
		**Locus^**†**^**	**% Identity**	**% Query coverage**	**Contig (length)**	**Predicted contig origin^**‡**^**
Ampicillin	Resistant	*bla*ORN^§^	99.89 (1 SNP)	100	Node 34 (60566)	Chromosome (0.947)
		*bla*TEM-1B	100	100	Node 87 (17794)	Plasmid (0.983)
Amoxicillin—clavulanic acid	Susceptible	*/*	*/*	/	*/*	*/*
Apramycin	Susceptible	*/*	*/*	/	*/*	*/*
Ceftiofur	Susceptible	*/*	*/*	/	*/*	*/*
Cefquinome	Susceptible	*/*	*/*	/	*/*	*/*
Doxycycline	Resistant	*tet*(D)	100	100	Node 128 (3634)	Plasmid (0.954)
Tetracycline	Resistant	*tet*(D)	100	100	Node 128 (3634)	Plasmid (0.954)
Enrofloxacin	Intermediate	*qnrS1*	100	100	Node 103 (10631)	Plasmid (0.967)
Florfenicol	Susceptible	*/*	*/*	/	*/*	*/*
Flumequine	Susceptible	*qnrS1*	100	100	Node 103 (10631)	Plasmid (0.967)
Gentamicin	Resistant	*aac(3)-IIa*	100	100	Node 87 (17794)	Plasmid (0.983)
Streptomycin	Resistant	*aph(3")-Ib*	99.88 (1 SNP)	100	Node 103 (10631)	Plasmid (0.967)
Spectinomycin	Susceptible	*/*	*/*	/	*/*	*/*
Sulfa-trimethoprim	Resistant	*sul2*	100	100	Node 103 (10631)	Plasmid (0.967)
		*dfrA14*	100	100	Node 38 (55589)	Plasmid (0.724)
Fosfomycin	Not tested	*fosA*	99.52 (2 SNPs)	100	Node 41 (50833)	Chromosome (0.994)

† Detected by both making an assembly with SPAdes and BLASTing, and direct read mapping with SRST2, against the ResFinder database containing a curated catalog of AMR genes.

‡ *Determined using mlplasmids. Between brackets the range of posterior probability is given. Klebsiella pneumoniae was used as species model, given it's close genetic relationship with Raoultella sp*.

§ *Gene is present as blaPLA (NCBI accession NG_049969.1) in the ResFinder database derived from R. planticola, but inspection demonstrated the isolate gene to correspond to blaORN (NCBI accession NG_049386.1—for which % identity and % query coverage values are presented) derived from R. ornithinolytica (see Supplementary Material)*.

### Treatment

The patient was suspected to have neonatal respiratory distress syndrome (NRDS) combined with sepsis. Based on the clinical presentation of the animal, therapy initialization could not be post-poned until microbial information was present. The calf was empirically treated with enrofloxacin (Floxadil® 5 mg/kg, Emdoka, Hoogstraten, Belgium), sodium benzylpenicillin (penicillin 1.500.000 IU q.i.d., Kela, Sint-Niklaas, Belgium) and oxygen (1.5 L/min.) for seven days, ketoprofen (Ketofen® 3 mg/kg, Ceva, Libourne, France) during the first 2 days and doxapram hydrochloride (Dopram® 1.5 mg/kg, Eumedica, Brussel, Belgium) on day one. Perfusions were given intravenously through a catheter, starting with bicarbonate 2% (20 gram bicarbonate in 1 liter of isotonic NaCl 0.9%; rate 100 mL/kg/h), followed by Ringer's lactate solution at a rate of 20 mL/kg/h. The calf received perfusions until it drank sufficiently on day 6. After ultrasonography and clinically improvement, the calf could leave the clinic.

## Follow-Up Diagnostics

### Whole Genome Sequencing

For research purposes, the isolated was further characterized by whole genome sequencing (WGS). Genomic DNA was prepared using the Isolate II Genomic DNA kit (Bioline, Meridian Bioscience, Paris, France), following the manufacturer's instructions. Sequencing libraries were constructed using the Illumina Nextera XT DNA sample preparation kit and subsequently sequenced on an Illumina MiSeq instrument with a 250-bp paired end protocol (MiSeq v3 chemistry) according to the manufacturer's instructions. Generated WGS data have been deposited in the NCBI Sequence Read Archive (SRA) (22) under accession number PRJNA607902. For species identification based on WGS data, three different approaches were used: k-mer based identification of read data using Kraken against the entire NCBI RefSeq Microbial Genomes database, 16S rRNA gene analysis using the NCBI suite of tools, and read mapping using Bowtie2 against the NCBI RefSeq reference genome entry for *R*. *ornithinolytica* (NCBI accession NC_021066.1) (22, 23). For genotypic antimicrobial resistance (AMR) gene detection, WGS data were searched using an in-house workflow based on assembly with SPAdes and BLASTing against the ResFinder database containing a curated catalog of AMR genes (24–27).

Mlplasmids 1.0.0 (https://sarredondo.shinyapps.io/mlplasmids) was used to determine whether the predicted contig origin was plasmid- or chromosome-derived. *Klebsiella pneumoniae* was used as species model. Additionally, a direct read mapping approach was performed using SRST2 against the ResFinder database. One of the detected isolate AMR genes, *bla*PLA, is a genetic marker that allows differentiation of *Raoultella* spp., and was further investigated using the NCBI suite of tools (7, 28). A more extended description of bioinformatics methods for WGS data analysis including tools, versions, and parameters, is available in the Supplementary Material.

Identification of the isolate as *R. ornithinolytica* was supported by WGS data through three different analyses. Firstly, k-mer based identification of WGS data against the NCBI RefSeq Microbial Genomes database classified 87.52% of reads as *R. ornithinolytica*, whereas no other species were detected using a 5% threshold (see Supplementary Figure 1). Secondly, 16S rRNA analysis using the NCBI RefSeq 16S database confirmed the presence of a 16S rRNA *R. ornithinolytica* gene with high sequence identity (>99%). Lastly, read mapping against the NCBI RefSeq Genome entry for *R. ornithinolytica* confirmed its presence with a depth and breadth of coverage of 30.52× and 94.52%, respectively (see Supplementary Figure 2). Genotypic AMR gene detection using the ResFinder database, both through an assembly- and read mapping-based approach, indicated the presence of several AMR genes consistently with both methods and various of the detected genes were predicted to be present on a plasmid (see Table 1). Isolate AMR genes that contained mutations compared to the ResFinder reference sequences were manually investigated. The isolate's *fosA* gene contained two synonymous mutations, and the isolate's *aph(3")-Ib* contained one mutation resulting in a K10E amino acid change, preserving the open reading frame of both isolate genes. The isolate's *bla*PLA gene, encoding a class A broad-spectrum beta-lactamase originally characterized from *R. planticola*, exhibited however 49 mutations. Because this gene acts as a species marker for *Raoultella* spp., the sequence of the isolate's AMR gene was further investigated, finding only one synonymous mutation compared to the *bla*ORN gene from *R. ornithinolytica*, confirming the functional presence of *bla*ORN and isolate identification as *R. ornithinolytica* (see Supplementary Figures 3, 4) (7). A more extended description of results for the WGS data analysis is available in the Supplementary Material.

## Discussion

To the authors' knowledge, this is the first report of a multidrug resistant *R. ornithinolytica*, isolated from a calf suffering from sepsis. Previously, *Klebsiella ornithinolytica* was retrieved from the viscera of a calf with suspected sepsis, but this sampling cannot be regarded evidence of involvement of these bacteria in sepsis (16). In addition, very recently, *R. ornithinolytica* has been identified in a presumed *Klebsiella* collection, associated with clinical mastitis in cattle (15). Indeed, based on biochemical characteristics, *Raoultella* was formerly incorrectly classified as *Klebsiella* (7, 29). Although conventional biochemical methods were not used in our case study, identification was confirmed both by MALDI-TOF MS and WGS. The increasing availability of MALDI-TOF MS in hospitals and diagnostic laboratories will likely result in a better and more frequent recognition of *R. ornithinolytica* in the years to come (6). On the other hand, this bacterium is likely underdiagnosed in septicemic animals, because blood culture enrichment is rarely used in veterinary practice and particularly not in food-producing animals, even though blood culture is the most appropriate sampling technique for presumed sepsis cases (1).

The calf most likely acquired the infection on the farm, since blood cultures were taken immediately upon arrival at the clinic. It cannot be excluded whether the intravenous treatment of the local veterinarian possibly caused the bacteremia as multiple venipuncture increases a higher risk of contamination (30). However, the authors deem this the be unlikely, since the calf was critically ill before ambulatory treatment. It is impossible to identify the portal of entry in this case. This animal also suffered from pneumonia, but whether the pneumonia was primary and sepsis the consequence, or whether the pneumonia resulted from septic spread is unclear. The ultrasonographic finding, with multiple consolidations and marked B-lines point more toward an interstitial or metastatic pneumonia than a classic bronchopneumonia.

The empirical antimicrobial choice was based on the most likely bacteria in bovine sepsis, namely *E. coli* and its local resistance profile, which is often multidrug resistant among others against tetracyclines and trimethoprim-sulfonamides (31). To maximize survival chances, as in human medicine, a de-escalation therapy, starting broad spectrum and narrowing the spectrum after microbial test results become available was anticipated (2). Sepsis in calves can also be of Gram positive origin, including anaerobes and *T. pyogenes* (32, 33). The choice fell on the combination of fluoroquinolones with sodium penicillin, because of the broad spectrum, rapid IV administration and bactericidal activity. In Belgium, the use of the critically important fluoroquinolones in food animals is legally regulated and taking an appropriate sample (in this case a blood culture) is mandatory (KB July 21st, 2016). Aminoglycosides could have been an alternative choice, but were not selected due to the risk for renal injury in especially in critically ill and dehydrated neonatal patients (34).

Despite the *R. ornithinolytica* isolate being phenotypically categorized as intermediately susceptible to enrofloxacin with disk diffusion, clinical response on enrofloxacin treatment combined with penicillin was good. The intermediate disk diffusion result is likely linked to the presence of the plasmid-mediated quinolone resistance (PMQR) gene *qnrS1*, which can indeed lead to low-level acquired resistance (Table 1) (35). Despite the clinical effectiveness of the treatment in this case, acquired resistance toward fluoroquinolones may result in treatment failure. Additionally, fluoroquinolones are critically important antibiotics for humans and should be used with caution and can be subject to local legislation. Nevertheless, veterinarians should be aware of the risk of multidrug resistance in *R. ornithinolytica* isolates, similar to the high resistance levels described in human medicine, where broad-spectrum antibiotic treatment is suggested before microbial test results are available (4).

AMR gene detection based on WGS data demonstrated the presence of several other AMR genes in the current isolate. *Raoultella* spp. are known to be intrinsically resistant against aminopenicillins due to the expression of a chromosomally encoded class A β-lactamase (36), which can explain its ineffectiveness when given by the ambulatory veterinarian. More specifically, for *R. ornithinolytica, bla*ORN has been described as a species marker and was also detected in our isolate (36). In addition, a *bla*TEM-1 β-lactamase gene predicted to be located on a plasmid was detected in the current isolate (Table 1), as described before (36). The presence of *bla*ORN and *bla*TEM-1 are in agreement with the phenotypic resistance observed against aminopenicillins. The current isolate was phenotypically resistant against gentamicin and streptomycin. This is in accordance with the presence of *aac(3)-II* and *aph(3")-I* encoding aminoglycosides modifying enzymes associated with resistance to gentamicin and streptomycin, respectively (Table 1) (37). Also for tetracyclines and potentiated sulphonamides, phenotypic resistance was supported by the presence of relevant resistance genes (Table 1).

For sepsis, broad-spectrum bactericidal drugs are recommended in human medicine (2, 38). Due to restrictions on the use of critically important antimicrobials, these crucial products for sepsis treatment are only conditionally available for (food) animals in some countries. Nevertheless, existence of multidrug resistant *R. ornithinolytica* might be a further motivation for veterinarians to apply diagnostics by blood culture to better target and rationalize antimicrobial therapy.

## Conclusion

This report raises the question whether *R. ornithinolytica* is currently underdiagnosed in veterinary medicine due to limited diagnostics, and whether it may be emerging in cattle similarly as recently documented for humans. Multidrug resistant *R. ornithinolytica* can apparently cause sepsis in calves similar to humans. Spread of such multidrug resistant pathogens would further hamper treatment options for sepsis. Clarification on its current prevalence, the potential existence of reservoirs in animals or farming environments, and the relationship with human isolates is warranted.

## Data Availability Statement

The datasets generated in this study can be found in online repositories. The names of the repository/repositories and accession number(s) can be found below: https://www.ncbi.nlm.nih.gov/, PRJNA607902.

## Ethics Statement

Ethical review and approval was not required for the animal study because it was a case description of research conducted for diagnostic purposes with the aim of a initializing a proper treatment for the patient. Written informed consent was obtained from the owners for the participation of their animals in this study.

## Author Contributions

All authors listed have made a substantial, direct and intellectual contribution to the work, and approved it for publication.

## Conflict of Interest

The authors declare that the research was conducted in the absence of any commercial or financial relationships that could be construed as a potential conflict of interest.

## Appendix

**Table A1:** Disk diffusion susceptibility for *Raoultella ornithinolytica* per antimicrobial agent, including information about potency disks, diameter control strains (*E. coli* ATCC 25922 and *E. coli* ATCC 35218) and associated quality control values, zone diameter *R. ornithinolytica* and values for interpreting a susceptibility category.

**Antimicrobial agent**	**Potency (μq)**	**Performance standards disk diffusion**	**CS *E. coli* ATCC 25922 zone diameter (mm) and QC ranges**	**CS *E. coli* ATCC 35218 zone diameter (mm) and QC ranges**	***R. ornithinolytica* zone diameter (mm)**	**Interpretive categories**			**Category susceptibility *R. ornitholytica***
						***S***	***I***	***R***	
Ampicillin	10	CLSI^a^	20 (15–22)	NA	8	≥17	14–16	≤13	Resistant
Amoxicillin—clavulanic acid	30 (A 20 C 10)	CLSI^a^	20 (18–24)	22 (17–22)	18	≥18	14–17	≤13	Susceptible
Enrofloxacin	10	Manufacturer^c^	32 (31–37)	NA	20	≥23	22–17	≤16	Intermediate
Flumequine	30	Manufacturer^c^	32 (28–36)	NA	20	≥20	19–17	≤16	Susceptible
Florfenicol	30	Manufacturer^d^	27 (22–28)	NA	24	≥20	19–17	≤16	Susceptible
Doxycycline	30	CLSI^a^	22 (18–24)	NA	8	≥14	11–13	≤10	Resistant
Tetracycline	30	CLSI^a^	22 (18–25)	NA	8	≥15	12–14	≤11	Resistant
Spectinomycin	200	Manufacturer^e^	26 (24–32)	NA	24	≥20	19–15	≤16	Susceptible
Apramycin	40	Manufacturer^c^	23 (21–28)	NA	26	≥23	22–20	≤19	Susceptible
Streptomycin	10	CLSI^a^	16 (12–20)	NA	8	≥15	12–14	≤11	Resistant
Gentamicin	10	CLSI^a^	20 (19–26)	NA	8	≥15	13–14	≤12	Resistant
Cefquinome	30	Manufacturer^c^	32 (28–36)	NA	32	≥23	22–20	≤19	Susceptible
Ceftiofur	30	CLSI^b^	26 (26–31)	NA	26	≥21	18–20	≤17	Susceptible
Trimethoprim—sulfamethoxazole	25 (T 1.25 S 23.75)	CLSI^a^	27 (23–29)	NA	8	≥16	11–15	≤10	Resistant

*NA, Not available; CS, Control strain; QC, Quality control; ATCC, American Type Culture Collection; S, Susceptible; I, Intermediate; R, Resistant. CLSI: Clinical and Laboratory Standards Institute (19)*.

a *Breakpoints specifically for Enterobacterales: Human*.

b *Breakpoints specifically for Enterobacterales: Cattle. Manufacturer: Rosco Diagnostica (20)*.

c *Breakpoints for slow growing organisms and other general bacteria species: Veterinary*.

d *Breakpoints for slow growing organisms and other general bacteria species: Cattle*.

e *Breakpoints for slow growing organisms and other general bacteria species: Human*.
