# Supplementary material for: Case Report: Multidrug Resistant Raoultella ornithinolytica in a Septicemic Calf
Source: Front Vet Sci. 2021 Mar 26;8:631716. doi: 10.3389/fvets.2021.631716 (PMC8032891; doi:10.3389/fvets.2021.631716)
Supplement: Supplementary file 1 [file Data_Sheet_1.docx]

Supplementary Material

# Analysis whole genome sequencing data

## Material and methods

### Isolate identification based on WGS data

Trimmomatic 0.36 (Bolger et al., 2014) was first used to trim raw reads setting the following options: ‘ILLUMINACLIP: NexteraPE-PE.fa:2:30:10’, ‘LEADING:10’, ‘TRAILING:10’, ‘SLIDINGWINDOW:4:20’, and ‘MINLEN:40’. Afterwards, trimmed reads were *de novo* assembled using SPAdes 3.13.0 (Bankevich et al., 2012) setting the following options: ‘-careful’, and ‘--cov-cutoff 10’. Orphaned reads resulting from trimming (i.e. reads where only one read of the pair survived) were also provided to the assembler as unpaired reads. Three different approaches were afterwards employed to confirm identification of *R. ornithinolytica*: k-mer based classification, 16S analysis, and comparison with the *R. ornithinolytica* reference genome.

For k-mer based identification, Kraken 0.10.6 (Wood & Salzberg, 2014) was used to perform k-mer based classification of cleaned reads against an in-house dump of the complete genomes from the NCBI RefSeq Microbial Genomes database (database retrieved 24/01/2018) (O'Leary et al., 2016). Results were then visualized with Krona (Ondov et al., 2011).

For 16S rRNA-based identification, the NCBI RefSeq 16S database was mined for 16S sequences of *R. ornithinolytica* using the query ‘((33175[BioProject]) OR 33317[BioProject]) AND "Raoultella ornithinolytica"[Organism]’, resulting in three partial 16S sequences (NCBI accessions NR_114736.1 (1,516 bp); NR_114502.1 (1,436 bp); and NR_044799.1 (1,502 bp)). The genome assembly was then compared using blastn (with default settings) hosted at NCBI against these three genes.

For comparison with the reference genome, the *R. ornithinolytica* reference genome was downloaded from NCBI RefSeq (NCBI accession NC_021066.1). Bowtie2 2.2.6 (Langmead et al.,, 2009) was then used to map trimmed paired-end reads setting the ‘--very-sensitive-local' option. PICARD 1.136 (available at <http://broadinstitute.github.io/picard/>) was then used to collect read coverage statistics using the ‘CollectWgsMetrics’ functionality setting the ‘MINIMUM_MAPPING_QUALITY="20"’, ‘MINIMUM_BASE_QUALITY="20"’, ‘COVERAGE_CAP="250"’, and ‘VALIDATION_STRINGENCY="LENIENT"’ options. Tablet (Milne et al., 2013) was afterwards used to visualize the read mapping results.

### Genotypic antimicrobial resistance gene detection

Genotypic resistance gene detection for the assembly using BLAST was performed as described by Bogaerts et al., 2019 against an in-house copy of the ResFinder database (Zankari et al., 2012) (database accessed 03/04/2019). Additionally, genotypic resistance gene detection based on direct read mapping against the ResFinder database was also performed using SRST2 0.2.0 (Inouye et al., 2014) using the following options: ‘--max_divergence 10’, ‘--gene_max_mismatch 10’, ‘--max_unaligned_overlap 150’, and ‘--min_coverage 90’. Results of both approaches are presented in Table 1 (main manuscript). Functional analysis of detected genes that did not have an identity of 100% were performed with Jalview 2 (Waterhouse et al., 2009).

### blaPLA analysis

One of the detected AMR genes, *bla*PLA*,* has recently been described as a genetic marker that allows to differentiate *R. ornithinolytica* (named *bla*ORN) from the closely related *R. planticola* (named *bla*PLA) (Ponce-Alonso et al., 2016). The sequence of the *bla*PLA gene in the isolate detected using the ResFinder database was therefore aligned using megablast (with default settings) hosted at NCBI against the nucleotide database, and the first 100 hits were retained and visualized using the TreeView Display with default options, for which FigTree (available at http://tree.bio.ed.ac.uk/software/figtree/) was used to visualize the resulting tree. Additionally, the detected isolate *bla*PLA gene was compared to reference genes from both *R. ornithinolytica* (NCBI accession NG_049386.1, *bla*ORN) and *R. planticola* (NCBI accession NG_049969.1, *bla*PLA) as described by Ponce-Alonso et al (Ponce-Alonso et al., 2016). MEGA 10.0.4 (Tamura et al., 2007) was used to align all three genes using the MUSCLE algorithm (with default settings). Overhangs on both ends of the alignment were trimmed, and then a minimum evolutionary tree was constructed with MEGA using default settings, which was visualized with FigTree.

## Results

### Isolate identification based on WGS data

K-mer based classification of reads from WGS data indicated that 87.52% of reads could be classified as *R. ornithinolytica* (see Figure S1). No other species were detected using a 5% threshold for the presence of a species.


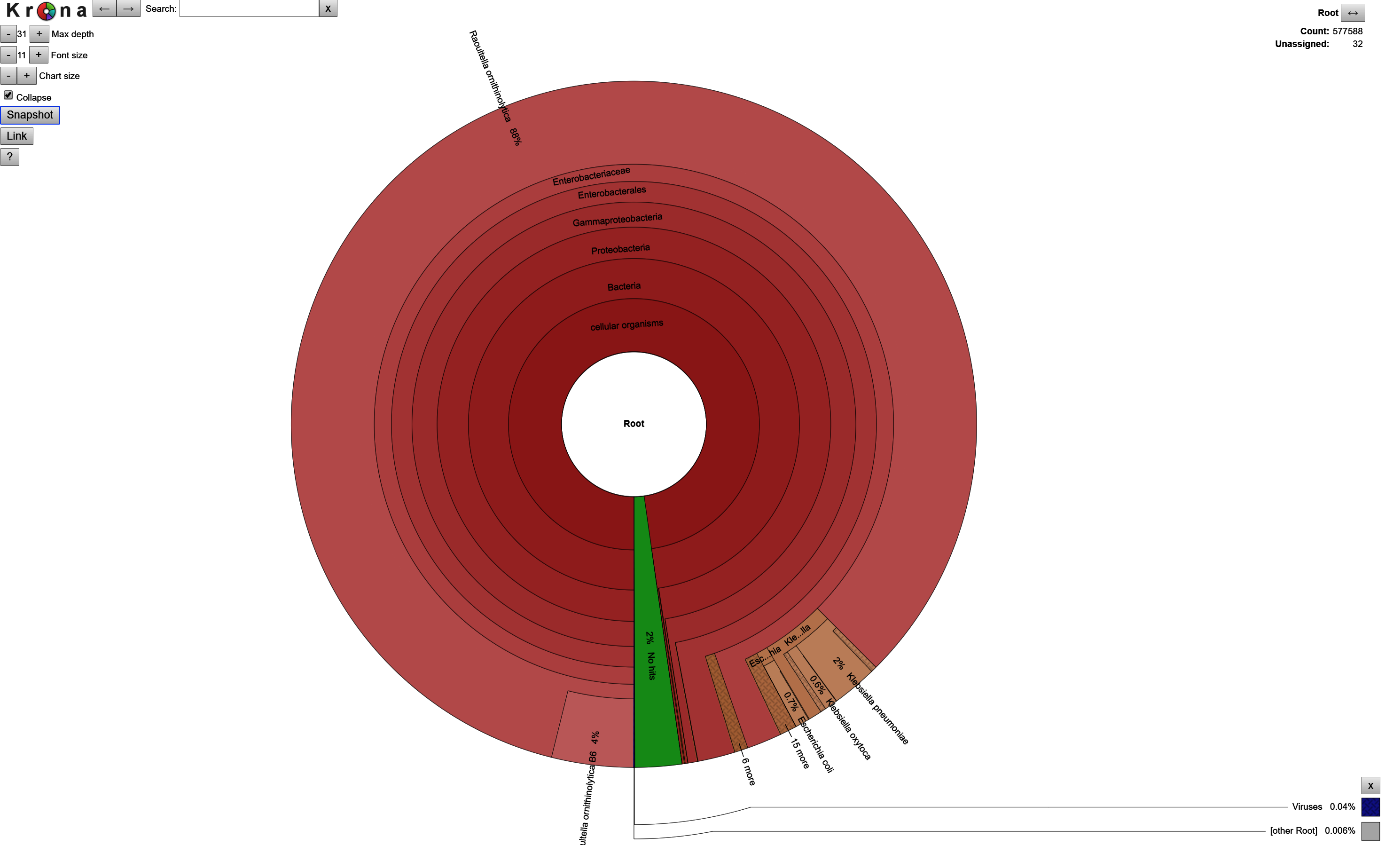


**Supplementary Figure 1.** K-mer based classification using Kraken confirms the identification of the isolate as *R. ornithinolytica.*

For 16S rRNA -based identification, comparison of all three 16S sequences available in NCBI RefSeq 16S database annotated as *R. ornithinolytica* resulted in full-length highly-identical (>99%) matches of the same locus in the assembly, supporting the classification as *R. ornithinolytica*.

For read mapping against the *R. ornithinolytica* NCBI RefSeq reference genome, the average depth, and breadth, were 30.52x and 94.52%, respectively. Visual inspection confirmed that the entire genome was covered by reads, with a more or less uniform distribution (see Figure S2).


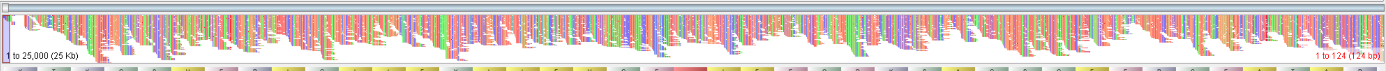


**Supplementary Figure 2.** Read mapping against the *R. ornithinolytica* reference genome demonstrates the presence of *R. ornithinolytica.*

Combined, these three analyses confirm that the sequenced isolate is *R. ornithinolytica*.

### Genotypic antimicrobial resistance gene detection

Results for genotypic AMR detection using both the assembly- and read mapping-based approaches are presented in Table 1 (main manuscript), and indicate resistance against the following classes of antibiotics: aminoglycoside, beta-lactam, trimethoprim, quinolone, sulphonamide, tetracycline, and fosfomycine. Functional effects on the coding sequences of isolate AMR genes for which not 100% identity with the ResFinder database was found, were individually investigated (results not shown). For *fosA*, two mutations were present in the isolate sequence that coded both for synonymous mutations resulting in the same coding sequence. For *aph(3’’)-Ib*, only one mutation in the isolate gene was present, resulting in an amino acid change at position 10 (K->E), and the Open Reading Frame (ORF) therefore remained intact. Lastly, for *bla*PLA, there were 49 mutations in total resulting in 274/291 identities and 282/291 similarities, although no frameshifts or stop codons were introduced. A more detailed investigation of this isolate AMR gene is described in the next section.

### blaPLA analysis

An explorative visualization of the isolate *bla*PLA gene detected with ResFinder compared to its first 100 hits in the NCBI nucleotide database is presented in Figure S3, and demonstrates that the isolate *bla*PLA gene clusters with other *R. ornithinolytica bla*ORN genes, clearly differentiated from another cluster carrying the *R. planticola* *bla*PLA genes. For the cluster containing the *R. ornithinolytica bla*ORN genes, only one *Klebsiella aerogenes* and one *R. planticola* hit are present that therefore most likely represent wrongly annotated genes in the NCBI nucleotide database.


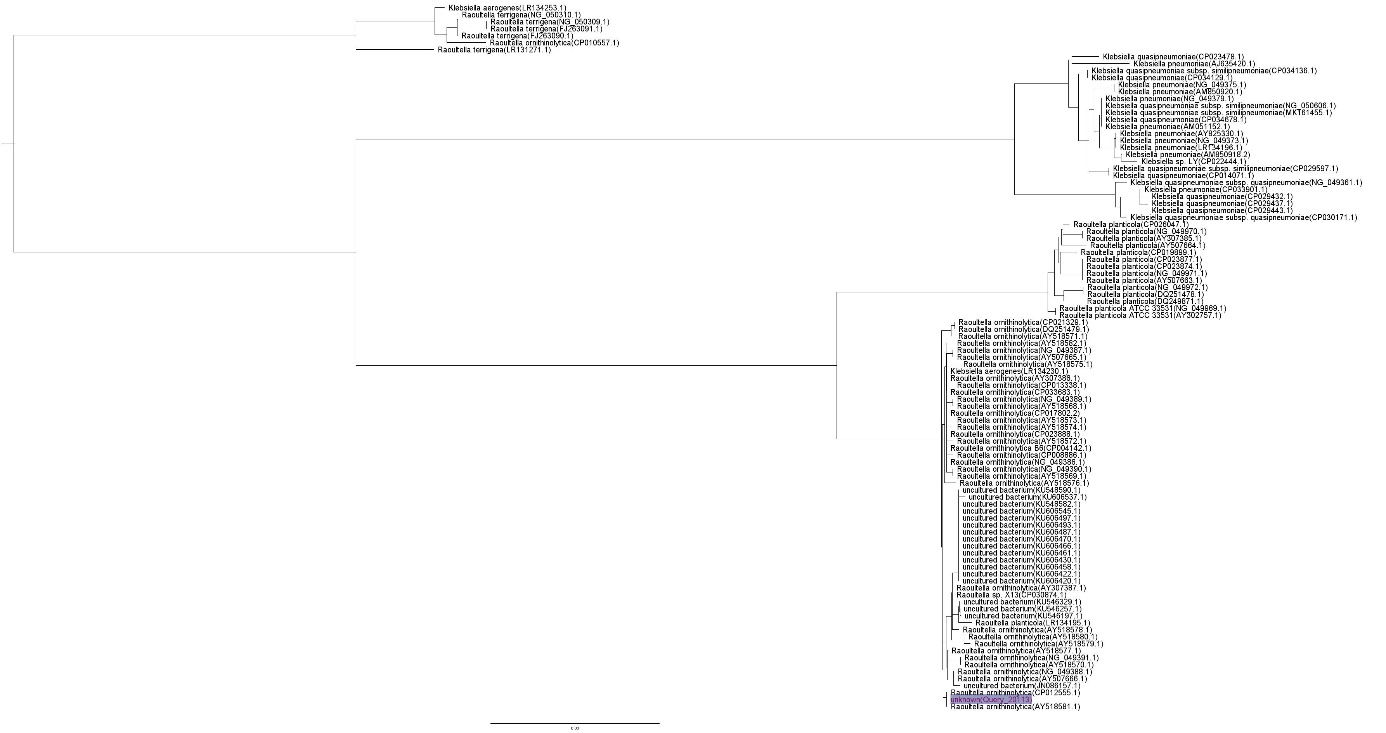


**Supplementary Figure 3.** Distance-based visualization of the isolate *bla*PLA gene (annotated in red as ‘unknownQuery_20113’) and the first 100 hits from the NCBI nucleotide database.

Results of comparing the isolate *bla*PLA gene with the coding sequences of two reference sequences for *R. ornithinolytica* (NCBI accession NG_049386.1, *bla*ORN) and *R. planticola* (Ponce-Alonso et al., 2016) (NCBI accession NG_049969.1, *bla*PLA) are presented in Figure S4. The isolate gene had only one mutation compared to the *R. ornithinolytica* *bla*ORN gene, but 49 mutations compared to the *R. planticola* *bla*PLA gene (Ponce-Alonso et al., 2016), and clustered consequently with the *R. ornithinolytica* gene. Moreover, the one mutation compared to the *R. ornithinolytica* *bla*ORN gene was a synonymous mutation resulting therefore in exactly the same ORF. The 49 mutations compared to the *R. planticola* *bla*PLA gene (Ponce-Alonso et al., 2016) were exactly the same as those found during the genotypic AMR detection against the ResFinder database (see above). Further inspection of the *bla*PLA gene in the ResFinder database indicated that the latter was indeed derived from the *R. planticola* genome (Ponce-Alonso et al., 2016).


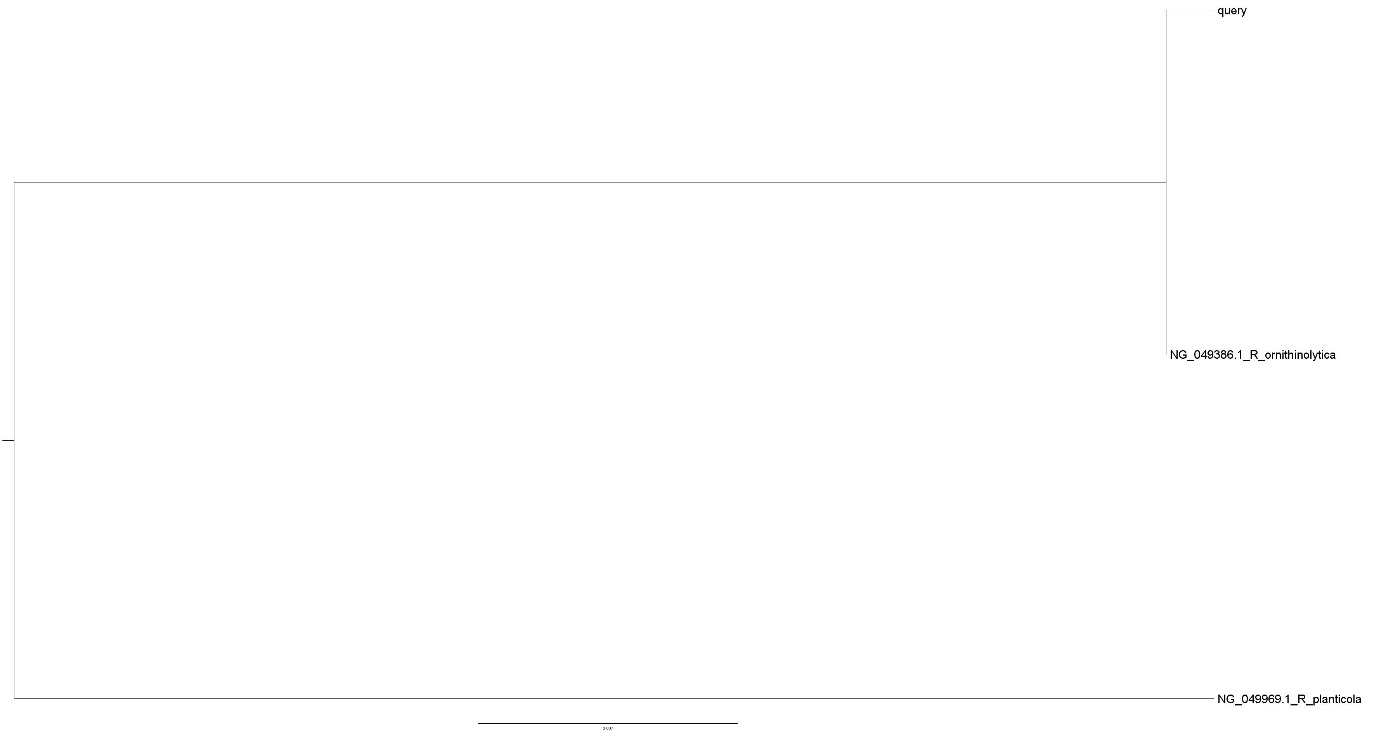


**Supplementary Figure 4.** Distance-based visualization of the isolate *bla*PLA gene (annotated as ‘query’) and the *R. ornithinolytica* *bla*ORN and *R. planticola bla*PLA genes (Ponce-Alonso et al., 2016).

These results support that the *bla*PLA gene detected in the isolate using the ResFinder database in fact constitutes *bla*ORN, which is functionally present. Moreover, this analysis confirms the identification of the isolate as *R. ornithinolytica* and not *R. planticola.*

## References

Bankevich, A., Nurk, S., Antipov, D., Gurevich, A. A., Dvorkin, M., Kulikov, A. S., et al. (2012). SPAdes: a new genome assembly algorithm and its applications to single-cell sequencing. *J Comput Biol, 19*(5), 455-477. doi:10.1089/cmb.2012.0021

Bogaerts, B., Winand, R., Fu, Q., Van Braekel, J., Ceyssens, P. J., Mattheus, W., et al. (2019). Validation of a Bioinformatics Workflow for Routine Analysis of Whole-Genome Sequencing Data and Related Challenges for Pathogen Typing in a European National Reference Center: Neisseria meningitidis as a Proof-of-Concept. *Front Microbiol, 10*. doi:ARTN 362 10.3389/fmicb.2019.00362

Bolger, A. M., Lohse, M., & Usadel, B. (2014). Trimmomatic: a flexible trimmer for Illumina sequence data. *Bioinformatics, 30*(15), 2114-2120. doi:10.1093/bioinformatics/btu170

Inouye, M., Dashnow, H., Raven, L. A., Schultz, M. B., Pope, B. J., Tomita, T., et al. (2014). SRST2: Rapid genomic surveillance for public health and hospital microbiology labs. *Genome Medicine, 6*. doi:ARTN 90 10.1186/s13073-014-0090-6

Langmead, B., Trapnell, C., Pop, M., & Salzberg, S. L. (2009). Ultrafast and memory-efficient alignment of short DNA sequences to the human genome. *Genome Biology, 10*(3). doi:ARTN R25 10.1186/gb-2009-10-3-r25

Milne, I., Stephen, G., Bayer, M., Cock, P. J. A., Pritchard, L., Cardle, L., et al. (2013). Using Tablet for visual exploration of second-generation sequencing data. *Briefings in Bioinformatics, 14*(2), 193-202. doi:10.1093/bib/bbs012

O'Leary, N. A., Wright, M. W., Brister, J. R., Ciufo, S., McVeigh, D. H. R., Rajput, B., et al. (2016). Reference sequence (RefSeq) database at NCBI: current status, taxonomic expansion, and functional annotation. *Nucleic Acids Research, 44*(D1), D733-D745. doi:10.1093/nar/gkv1189

Ondov, B. D., Bergman, N. H., & Phillippy, A. M. (2011). Interactive metagenomic visualization in a Web browser. *BMC Bioinformatics, 12*. doi:Artn 385 10.1186/1471-2105-12-385

Ponce-Alonso, M., Rodriguez-Rojas, L., del Campo, R., Canton, R., & Morosini, M. I. (2016). Comparison of different methods for identification of species of the genus Raoultella: report of 11 cases of Raoultella causing bacteraemia and literature review. *Clinical Microbiology and Infection, 22*(3), 252-257. doi:10.1016/j.cmi.2015.10.035

Tamura, K., Dudley, J., Nei, M., & Kumar, S. (2007). MEGA4: Molecular evolutionary genetics analysis (MEGA) software version 4.0. *Molecular Biology and Evolution, 24*(8), 1596-1599. doi:10.1093/molbev/msm092

Waterhouse, A. M., Procter, J. B., Martin, D. M. A., Clamp, M., & Barton, G. J. (2009). Jalview Version 2-a multiple sequence alignment editor and analysis workbench. *Bioinformatics, 25*(9), 1189-1191. doi:10.1093/bioinformatics/btp033

Wood, D. E., & Salzberg, S. L. (2014). Kraken: ultrafast metagenomic sequence classification using exact alignments. *Genome Biology, 15*(3). doi:ARTN R46 10.1186/gb-2014-15-3-r46

Zankari, E., Hasman, H., Cosentino, S., Vestergaard, M., Rasmussen, S., Lund, O., et al. (2012). Identification of acquired antimicrobial resistance genes. *Journal of Antimicrobial Chemotherapy, 67*(11), 2640-2644. doi:10.1093/jac/dks261
